# Comparative volatile composition, antioxidant and cytotoxic evaluation of the essential oil of *Zhumeria majdae *from south of Iran

**DOI:** 10.22038/ijbms.2018.20829.5418

**Published:** 2019-01

**Authors:** Mansoor Saeidi, Javad Asili, Seyed Ahmad Emami, Nasrin Moshtaghi, Saeid Malekzadeh-Shafaroudi

**Affiliations:** 1 Department of Biotechnology and Plant Breeding, Faculty of Agriculture, Ferdowsi University of Mashhad, Mashhad, Iran; 2 Department of Pharmacognosy, School of Pharmacy, Mashhad University of Medical Sciences, Mashhad, Iran

**Keywords:** Antioxidant activity, Cytotoxicity, Essential oil, Lamiaceae, *Zhumeria majdae*

## Abstract

**Objective(s):**

The purpose of this study was to evaluate variations in yields, volatile composition and biological activities of essential oils (EOs) obtained from the aerial parts of *Zhumeria majdae *collected from five localities of the south of Iran.

**Materials and Methods:**

The EOs were analyzed using gas chromatography and gas chromatography-mass spectrometry techniques. The antioxidant activity of the EOs was tested using DPPH and β-carotene/linoleic acid assays. *In vitro* cytotoxicity was tested against two cancer cell lines (A375 and MCF7) using MTT assay.

**Results:**

The oils yield varied from 6.3% (S2) to 10.2% (V/W) (S4). All of five investigated EOs samples presented three major compounds: linalool (24.4-34.6%), camphor (26.1-34.7%) and trans-linalool oxide (7.6-28.6%). Although the main constituents were common, their percentages were different. Among samples, S1 had a better antioxidant activity in both DPPH and β-carotene/linoleic acid methods (IC_50_= 8.01 and 11.77 mg/ml, respectively). *In vitro* cytotoxicity against two cancer cell lines of human melanoma cell line (A375) and breast cancer cell line (MCF7), showed a moderate cytotoxicity of S3 against A375 cells with IC_50_ value of 624 μg/ml.

**Conclusion:**

Tangezagh (S4) plant materials revealed the highest level of oil yield as the region is recommended for collecting the plant samples.Taken together, despite the weak antioxidant and moderate cytotoxic activities of tested EOs, this study suggested a proper potential for possible use of the EOs of *Z. majdae* for pharmaceutical and perfume industries.

## Introduction

*Zhumeria majdae *is a perennial fragrant shrub, belongs to Lamiaceae (Labiatae) family, endemic to the southern parts of Iran that grows on rather bare rocky slopes (1, 2). Its EO has a strong pleasant odor. Essential oils are one of the main sources of biologically active compounds (3). For some time, this plant have been used as a curative for stomach aches, flatulence, diarrhea, indigestion, cold, headache, wound healing and as antiseptic and treatment of painful menstruation (4). Phytochemically, the presence of some compounds such as flavonoids, diterpenoids and triterpenoids in *Z. majdae *have revealed (5, 6). Cytotoxic, antileishmanial and antiplasmodial activities of 12,16-dideoxy aegyptinone B from *Z. majdae *were reported (7)**. **There are reports about the anti-inflammatory (8), antinociceptive, acute toxicity (9) and anticonvulsant (10) activities of the EO and extract of *Z. majdae*. In case of antimicrobial activity, the EO of *Z. majdae *was more active on *Escherichia coli* than *Staphylococcus aureus *(11)**. **In another study, its EO showed high antimicrobial activity against *Staphylococcus epidermidis*, *Bacillus pumulis* and *Bacillus subtilis *(12). Previous studies on the volatile composition of *Z. majdae *EO have shown the high levels of linalool (35.6-53.3%) and camphor (23.8-43.0%) (13-14). Our current study, the cytotoxic and antioxidant activity of *Z. majdae* EO was reported for the first time. Previous study showed that light, day length, mineral nutrients, drought, light intensity and altitude affected plants EO content (15). However, according to our knowledge, no comparative study has been published on volatile composition and biological activity of *Z. majdae *with respect to the impact of geographic variation. Therefore, the purpose of this work was to evaluate the effect of different environmental conditions on yield, volatile composition, antioxidant and cytotoxic properties of *Z. majdae *EO.

## Materials and Methods

***Chemicals and solvents***

2,2-Diphenyl-1-picrylhydrazyl (DPPH), β-carotene, linoleic acid, butylated hydroxyl toluene (BHT) and vit C were purchased from Sigma–Aldrich (Steinheim, Germany). All solvents as analytical grade were purchased from Dr Mojallali Lab (Tehran, Iran).

***Plant materials***

The aerial parts of *Z. majdae *were collected during its flowering stage at five locations from south of Iran: Sirmand; S1 (Voucher Herbarium number: 2-1812), Ghotbabad; S2 (No 2-1813), Sarchahan; S3 (No 2-1814), Tangezagh; S4 (No 2-1815), and Geno; S5 (No 2-1816), in May 2014 (Table 1). The climate of collection area is represented by warm to hot, temperate summer and moderate winters. Voucher specimens were prepared and identified by Mr MR Joharchi and deposited at the Herbarium of the Department of Pharmacognosy in Mashhad University of Medical Sciences, Mashhad, Iran.

***Isolation of EO***

EOs were extracted by hydro-distillation of plant materials using a Clevenger-type apparatus (3 hr). The yellow oils were dried over anhydrous sodium sulphate.

***GC and GC–MS analysis***

Gas chromatography was carried out using Varian CP-3800 system equipped with a flame ionization detector (FID) and a CP-Sil 8CB fused-silica column (50 m × 0.25 mm, film thickness 0.12 m). The injector temperature and detector temperature were 260 and 280 ^°^C, respectively. The initial GC oven temperature was held at 50 ^°^C for 5 min, then increased up to 250 ^°^C with 3 ^°^C /min and held constant at 250 ^°^C for 10 min. The split ratio was used at 1:5, with the carrier gas, N2 (2 ml/min).

A quadruple mass detector and a HP-5 MS column (30 m × 0.25 mm I.D., film thicknesses 0.25 µm) were applied for the GC–MS analyzes. The oven temperature was kept at 50 ^°^C for 5 min and increased from 50^°^C to 250^°^C at a rate of 3 ^°^C/min and held at 250 ^°^C for 10 min. Other analytical settings were: injector temperature of 250 ^°^C; injection volume of 0.1 µl in split mode 1:50; carrier gas: Helium at 1.1 ml/min; ionization potential: 70 eV; ionization current: 150 µA; and mass range: 35-465. The compounds were based on the comparison of retention indices (RI) relative to n-alkanes, retention time (RT) and mass spectra. Library search was carried out using the Wiley 7n.L spectral database as well as by co comparing with the mass spectral data with those reported in the literature (16). Quantification of the relative amount of the each constituent was done according to the area under the curve method without consideration of calibration factor (17).

***Antioxidant activity***

*DPPH radical scavenging*

The free radical scavenging activity of EOs was evaluated by radical scavenging (DPPH) activity evaluation method (18). Two and half ml of the EOs (EO+MeOH) at different concentrations (40-1.25 mg/ml) was added to one ml of a DPPH methanol solution. After 30 min of incubation at room temperature, the absorbance was read at 518 nm. The radical scavenging activity of EOs was calculated from the equation: 

AA % = 100 – [(Abs _sample_ – Abs _blank_) / Abs _control_ ] × 100 

Methanol plus EOs was used as blank. DPPH solution plus methanol was used as a negative control. The positive controls were those using the standard solutions. Butyl hydroxyl toluene (BHT) and vitamin C (Vit C) were used as positive controls. All experiments were done in triplicate

*β-Carotene/linoleic acid (BCB) assay*

The BCB assay was performed according to the standard method, with slight modifications (19). Eight mg of β-carotene was dissolved in 8 ml chloroform. One ml the carotene-chloroform solution was added to 20 mg linoleic acid and 100 mg Tween 40. Chloroform was removed using a rotary evaporator and oxygenated distilled water was added to the residue and the mixture was sonicated for 1 min to form emulsion A. The emulsion B (20 mg linoleic acid, 200 mg Tween 40 and 50 ml water) was also prepared. Two hundred μl of EO (final concentrations: 40-1.25 mg/ml) was added to tubes containing 5 ml of the emulsion A. The absorbance was read at 0 min and after 120 min of incubation at 470 nm. Antioxidant activity was expressed as inhibition percentages of the samples which calculated from the equation:

100× (A_c (0)_ – A_c (120) _)/(A_s (120)_ – A_c (120) _) = %I 

Where A_s (120)_ is the absorbance of the sample at t=120 min, A_c (120)_ is the absorbance of the control at t=120 min, and A_c (0)_ is the absorbance of the control at t = 0 min. All experiments were done in triplicate.

*Cell culture*

A375 (human melanoma cell line) and MCF7 (human breast cancer cell line) were obtained from National Cell Bank of Iran (Pasteur Institute, Tehran, Iran). Cell lines were maintained in RPMI 1640 medium supplemented with 10 % (v/v) fetal bovine serum (FBS) (Gibco, Invitrogen, Paisely, UK), penicillin (100 units/ml) and streptomycin (100 mg/ml). Cells were incubated at 37 ^°^C under 5% CO_2_/95 % air in a humidified atmosphere.

*Cytotoxicity assay*

Cytotoxicity of the EOs was measured using MTT assay. Briefly, 5×10^3^ of MCF7 and A375 cells were seeded in a 96-well plate. After waiting 24 hr for adhesion, a serial of double diluted EOs (1600-50 μg/ml) was added to triplicate wells. After 48 hr, 10 µl of MTT solution (5 mg/ml in PBS) was added and the plates were incubated at 37 ^°^C for an additional 4 hr. The supernatant was then discarded and 200 µl DMSO was added to the culture to dissolve the formazan blue. Cytotoxicities were expressed as the concentration of a certain drug that inhibits cell growth by 50% (IC_50_). 

## Results

***Chemical composition of the EOs***

The present study has evaluated the effect of environmental conditions on the chemical composition and biological activities of the EOs of *Z. majdae *were collected from five localities in the south of Iran. The yields of EO ranged from 6.3% (S2) to 10.2% (S4) (V/W), calculated on the dry weight. Identified constituents of the EOs of *Z. majdae *are shown in Table 2.

***Antioxidant activity***

The antioxidant activities of the EOs of *Z. majdae *samples (S1-S5) were tested using DPPH and β-carotene/linoleic acid assay methods. Results are presented in Table 3. 

***Cytotoxicity assay***

Cytotoxicity of the EOs was measured on two human cancer cell lines; A375, and MCF7 using the MTT assay. The cell lines were subjected to increasing doses of the EOs ranging from 12-1600 μg/ml. After exposure to the EOs of *Z. majdae* for 48 hr, the growth of cell lines was inhibited in a concentration-dependent manner (Table 4).

## Discussion

***Chemical composition of the EOs***

The quality and industrial values of the EOs is mainly determined on the basis of amount of EO and the presence of some major bioactive compounds in it. Therefore, finding the optimal conditions for obtaining the best quality essential oil is necessary for commercial purposes. Plant EO quality and quantity are the result of interactions between several internal and external factors including; plant tissues used for extraction, maturity of the plant parts, growth stages, altitude, irrigation, seasonal variation, genetic variation, stress experienced by the plant due to soil and environmental factors (20). Also, the influences of geography and environment on the plant secondary metabolites which could be due to the effects on the biosynthesis pathways, were reported in many plants (21). In this study, it should be noted that hydrodistillation of *Z. majdae* aerial parts afforded a large amount of EO. Thirty eight components were identified in the S1 EO representing 98.4%, which is mainly composed of oxygenated monoterpenes, represented exclusively by linalool (29.6%), camphor (27.4%), trans-linalool oxide (18.7%) and limonene (3.7%). The S1 EO yield was 9.1% (v/w). Thirty six constituents representing 98.3% were identified in the S2 EO which trans-linalool oxide (28.6%), camphor (27.2%), linalool (24.4%) and limonene (3.4%) were found to be the main components. The S2 EO yield was obtained 6.0% (v/w). Aromadendrene, α-thujene and thymol did not found in the S2 EO while α-humulene and 3-car-3-ene-2-one were only presented in the same one. Thirty seven constituents representing 97.8% in the S3 EO, were composed mainly of linalool (34.2%), camphor (27.7%), trans-linalool oxide (16.2%) and limonene (2.6%). The S3 EO yield was 8.8% (v/w). Similar results with slightly higher levels of limonene (3.8%) and EO yield (10.2%, v/w) were reported for S4 EO. In this sample, 39 constituents representing 98.3% were identified with linalool (33.9%), camphor (26.1%) and trans-linalool oxide (14.6%) as the main constituents. Thirty six constituents representing 98.2% detected in the S5 EO that camphor (34.7%), linalool (34.6%), trans-linalool oxide (7.6%) and borneol (3.4%) were the main compounds. The S5 EO yield was obtained 7.4% (V/W).

The highest amount of camphor and linalool was found in the EO of S5. Also the lowest amount of trans-linalool oxide was detected in S5 EO (7.5%). The results revealed that the yields of EOs (6.0-10.2%) could be affected by environmental and geographical conditions. In all the investigated samples (S1-S5) oxygenated monoterpenes were identified as the main class of compounds, in agreement with previous reports, except for trans-linalool oxide (13, 22). In this study, some components such as car-3-en-2-one (1.7%) and α-humulene (trace) were found just in sample S2, while α-thujene, thymol and aromadendrene were not detected in S2 whilst they were detected in other samples. According to the results of this research, some constituents like *trans*-linalool oxide, though present in all samples exhibit significant quantitative variation, however, there were no significant differences among samples in point of main constituents, but in different percentages. By our data it is difficult to highlight any conclusive trend relating to qualitative oil chemical composition with environmental conditions for *Z. majdae *in tested samples.

**Table 1:** Some geographical characteristics of the collected *Z. majdae* samples in south of Iran

**Geographical characteristic**	**S1**	**S2**	**S3**	**S4**	**S5**
**Latitude**	N 27^°^ 58^´^	N 27^°^ 46^´^	N 27^°^ 57^´^	N 27^°^ 56^´^	N 27^°^ 26^´^
**Longitude**	E 56^°^ 3^´^	E 56^°^ 4^´^	E 55^°^ 5^´^	E 55^°^ 57^´^	E 56^°^ 17^´^
**Altitude (m)**	1355	733	1094	1128	349
**Mean yearly temperature(** **°C)**	15-17.5	21-23	17.5-20	20-22.5	25-27.5
**Rainfull (mm/year)**	325-350	280-300	300-325	290-310	275-300

**Table 2:** Chemical composition variability of five EOs samples from the aerial parts of *Z. majdae*

No.	Compounds	RI	Percentage				
			S1	S2	S3	S4	S5
1	Tricyclene	926	0.1	0.1	0.1	0.1	0.1
2	α-Thujene	930	*t*	*t*	0.1	*t*	*t*
3	α-Pinene	936	1.1	0.8	1.0	1.1	0.8
4	Camphene	952	2.6	2.2	2.6	2.9	2.3
5	Sabinene	976	*t*	-	*t*	*t*	-
6	β-Pinene	977	0.1	*t*	0.1	0.1	0.1
7	3-Octanone	990	0.5	0.8	0.9	1.0	0.7
8	Myrcene	993	0.4	0.2	0.3	0.3	0.2
9	α-Terpinene	1017	0.2	0.1	-	0.2	0.2
10	ρ-Cymene	1027	0.9	0.1	1.1	0.7	0.2
11	Limonene	1029	3.7	3.4	2.6	3.8	1.7
12	*cis-*Ocimene	1043	*t*	*t*	*t*	*t*	*t*
13	β-Ocimene	1053	0.1	0.1	0.1	0.1	0.1
14	γ-Terpinene	1062	0.5	0.2	0.6	0.5	0.4
15	*cis*-Linalool oxide	1076	0.9	1.0	0.9	0.9	0.9
16	Terpinolene	1089	0.9	0.2	1.0	1.0	1.0
17	*trans*-Linalool oxide	1113	18.7	28.6	16.2	14.6	7.6
18	Linalool	1129	29.6	24.4	34.2	33.9	34.6
19	Camphor	1160	27.4	27.2	27.7	26.1	34.7
20	Borneol	1173	1.8	2.3	2.1	2.6	3.4
21	*trans*-β-Terpineol	1183	0.9	0.8	-	0.9	1.0
22	α-Terpineol	1195	1.1	0.9	1.0	1.1	1.3
23	Verbenone	1207	0.1	0.1	0.1	0.1	0.1
24	*cis*-Carveol	1222	0.1	-	0.1	0.1	-
25	Nerol	1232	0.2	0.4	0.3	0.6	0.8
26	*cis-*p-Menta-1(7),8-dien-2-ol	1234	0.3	0.2	0.3	0.2	0.2
27	Neral	1246	0.8	0.7	0.7	0.7	0.7
28	Car-3-en-2-one	1253	-	1.7	-	-	-
29	Geraniol	1264	1.9	*t*	1.5	1.8	1.8
30	Geranial	1276	1.2	0.8	0.9	1.1	0.8
31	Limonen-10-ol	1292	0.2	0.1	0.1	0.2	0.2
32	Thymol	1296	0.1	-	0.1	0.1	*t*
33	Eugenol	1360	*t*	*t*	*t*	*t*	*t*
34	*trans*-Jasmone	1398	0.2	*t*	0.2	0.2	0.3
35	Dodecanal	1410	*t*	*-*	*t*	*t*	*-*
36	*trans*-Caryophyllene	1417	0.4	0.2	0.2	0.4	0.9
37	α-Humulene	1452	-	*t*	-	-	-
38	Aromadendrene	1550	*t*	*-*	*t*	*t*	*t*
39	Caryophyllene oxide	1582	1.1	0.7	0.8	0.8	1.2
40	α-Eudesmol	1509	0.2	0.1	0.1	0.2	0.2
41	7-epi-α-Eudesmol	1651	*-*	*t*	*t*	*t*	*t*
42	Major Grouped Compounds						
	Monoterpene hydrocarbons		10.6	7.3	9.4	10.8	6.9
	Oxygenated monoterpenes		85.5	89.1	86.3	85.0	88.2
	Sespuiterpene hydrocarbons		0.4	0.2	0.3	0.4	0.9
	Oxygented sesquiterpenes		1.3	0.9	0.9	1.0	1.4
	Miscellaneous compounds		0.6	0.8	0.9	1.1	0.8
	Total identified		98.4	98.3	97.8	98.3	98.2

Note: RI: Retention indices on CP-Sil 8CB capillary column relative to C_8_-C_20_ n-alkanes. *t*: trace <0.05%

**Table 3:** Total antioxidant potential (IC_50_, μg/mL), by DPPH and BCB methods for the EOs of *Z. majdae* samples collected from different locations

**Sample**	**(DPPH)** **IC**_50_**(mg/mL)**	**(BCB)** **IC**_50_**(mg/mL)**
**S1**	8.01±0.12	11.77±0.17
**S2**	8.79±0.13	13.65±0.23
**S3**	14.21±0.21	21.31±0.22
**S4**	9.62±0.22	15.50±0.26
**S5**	18.47±0.26	29.82±0.32
**BHT**	0.013±0.00	0.016±0.00
**Vit C**	0.009±0.00	0.011±0.00

**Table 4:** Cytotoxic activity of the essential oils of *Z. majdae* samples

**IC**_50_ ** (µg/mL)**					**Cell lines**
**S5**	**S4**	**S3**	**S2**	**S1**	
718.8±9.2	779.5±9.7	624.6±7.6	666.1±8.3	746.6±8.8	**A375**
642.7±7.8	646.9±9.5	732.3±10.3	717.9±6.2	674.3±5.5	**MCF7**

***Antioxidant activity***

Although the two tests yielded quantitatively different values, the best antioxidant activity was observed for S1 with IC_50_ value of 8.01 mg/ml, followed by S2 with IC_50_ value of 8.79 mg/ml, in DPPH method. The EO of S5 showed the lowest antioxidant activity with IC_50_ value of 18.47 mg/ml. This activity was lower in comparison with the antioxidant effect of BHT and vit C (IC_50_= 0.013 and 0.009 mg/ml, respectively). Previous study on the antioxidant activity of the ethylacetate sub-fraction of *Z. majdae* extract showed an IC_50_= 41.85 µg/ml, more effective than the *Z. majdae *EO in DPPH method. This potent activity may be attributed to the presence of high phenolic compounds specially quercetinin in ethylacetate sub-fraction (23). In the β-carotene/linoleic acid test, IC_50 _values of the EOs from the *Z. majdae* on the oxidation of the β-carotene in the presence of linoleic acid oxidation intermediates ranged from 11.77 to 29.82 mg/ml. The EOs also demonstrated weak antioxidant activity in this test. The order of activity was as follows: S1> S2> S4> S3> S5. The highest activity was observed for S1 with IC_50_ value of 11.77 mg/ml followed by S2 with IC_50 _value of 13.65 mg/ml. The lowest activity was observed for S5 with IC_50_ value of 29.82 mg/ml. The β-carotene bleaching activity of EO was weaker than that of the positive controls BHT and vit C (IC_50_ = 0.016 and 0.011 mg/ml, respectively). In both DPPH and β-carotene bleaching assays, the S1 and S2 showed better antioxidative capacity than the others. Some authors demonstrated that a significant linear correlation between total phenol content and antioxidant capacity (24). According to our analysis of the chemical composition of the EO, its low phenolic content could be responsible for its weak antioxidant activity. However, EOs are very complex and this property makes it difficult to explain the antioxidant properties, so it is difficult to attribute the antioxidant activity of the EO to one or some active compounds. Both minor and major compounds could make a significant contribution to the activity (25).

***Cytotoxic assay***

In our study, there was no considerable cytotoxicity variability between the examined geographical locations. A375 and MCF7 were most sensitive to the S3 and S5 with IC_50_ values of 624 and 642 μg/ml, respectively. On the whole, the EOs of *Z. majdae *samples exhibited moderate cytotoxicity. This study is the first report that describes the cytotoxic activity of the EO of the *Z. majdae, *but according to data available in the literature, it could be hypothesized that camphor and linalool may be attributed to account for the cytotoxic activity of the *Z. majdae *EO. Linalool, as one of the main component of *Z. majdae* EO, has been reported to be cytotoxic to C32 and ACHN (26). It was demonstrated that linalool can have cytotoxic effects by inducing cells to undergo apoptosis and triggering cell death (27). However, in another study, linalool was not active against PC-3, MDA-MB-231, Hs 578T, MCF7, SK-MEL-28, and 5637 human tumor cells in concentration of 100 μg/ml (28). In addition, the non-cytotoxic effect of camphor and borneol was reported (29, 30). However, minor components may be contribute to the cytotoxicity of the oil. It is also possible that the minor constituents may be involved in some type of synergism with the other active compounds, which deserves attention in future studies.

## Conclusion

The results presented in this work are the first report on the variation on yields, chemical composition, antioxidant and cytotoxic activities of EOs from *Z. majdae *collected from five localities in the south of Iran. Our results showed that the geographical variation did not have significant effect on major components of EOs except for trans-linalool oxide (7.5-28.5%). On the basis of findings, for achieving the highest oil yield (10.2%), the plant material should be collected from the Tangezagh (S4) region, in which possesses great economic value. With respect to antioxidant activity, results indicated some variation between samples. Although the results of the present study demonstrated weak antioxidant and moderate cytotoxic activities for the tested EOs, these activities suggested a proper potential for possible use of the EOs of *Z. majdae *for pharmaceutical and perfume industries. In addition to its activity, considering its high yield in EO, pleasant smell, and safety in bioactive concentrations, it can be also introduced as an industrial potential interest.
